# Integrated Health Risk Assessment of Heavy Metals in Suxian County, South China

**DOI:** 10.3390/ijerph120707100

**Published:** 2015-06-24

**Authors:** Daping Song, Dafang Zhuang, Dong Jiang, Jingying Fu, Qiao Wang

**Affiliations:** 1 Key Lab for Resources Use and Environmental Remediation, Institute of Geographical Sciences and Natural Resources Research, Chinese Academy of Sciences, 11A, Datun Road, Chaoyang District, Beijing 100101, China; E-Mails: songdp@lreis.ac.cn (D.S.); zhuangdf@igsnrr.ac.cn (D.Z.); fujy@lreis.ac.cn (J.F.); 2 Satellite Environmental Application Center, Ministry of Environmental Protection, Beijing 100094, China; E-Mail: wangqiao@sepa.gov.cn

**Keywords:** mining activities, heavy metals, soil and crop contamination, risk assessment, spatial distribution

## Abstract

The purpose of this study was to assess soil heavy metal contamination and the potential risk for local residents in Suxian county of Hunan Province, southern China. Soil, rice and vegetable samples from the areas near the mining industrial districts were sampled and analyzed. The results indicate that the anthropogenic mining activities have caused local agricultural soil contamination with As, Pb, Cu and Cd in the ranges of 8.47–341.33 mg/kg, 19.91–837.52 mg/kg, 8.41–148.73 mg/kg and 0.35–6.47 mg/kg, respectively. GIS-based mapping shows that soil heavy metal concentrations abruptly diminish with increasing distance from the polluting source. The concentrations of As, Pb, Cu and Cd found in rice were in the ranges of 0.02–1.48 mg/kg, 0.66–5.78 mg/kg, 0.09–6.75 mg/kg, and up to 1.39 mg/kg, respectively. Most of these concentrations exceed their maximum permissible levels for contaminants in foods in China. Heavy metals accumulate to significantly different levels between leafy vegetables and non-leafy vegetables. Food consumption and soil ingestion exposure are the two routes that contribute to the average daily intake dose of heavy metals for local adults. Moreover, the total hazard indices of As, Pb and Cd are greater than or close to the safety threshold of 1. Long-term As, Pb and Cd exposure through the regular consumption of the soil, rice and vegetables in the investigated area poses potential health problems to residents in the vicinity of the mining industry.

## 1. Introduction

In the course of the exploitation of heavy metal mining, a mining area and its surrounding environment may become affected by serious pollution from heavy metals. Emissions of heavy metals can contaminate groundwater and surface water, agricultural soils, and food crops; they also pose a health risk to residents near mining areas [1,2]. Since 1970, mining activities entered a period of rapid development worldwide. Numerous reports indicate that water, soil, vegetables and dust have been heavily polluted by lead (Pb), arsenic (As), copper (Cu), chromium (Cr), zinc (Zn) and cadmium (Cd) near the mining areas [3,4,5,6]. Pb, As, Cu, Cr and Cd are important toxic heavy metals, and have been identified as health risks by World Health Organization (WHO) [7,8]. South China has encountered serious environmental problems posed by these heavy metals in recent years. Liu, *et al.* [3] noted that the mean concentrations of Pb, As and Cd in the soils of Chenzhou city in South China were 751.98 mg/kg, 459.02 mg/kg, and 6.77 mg/kg, respectively. Zhuang, *et al.* [9] concluded that the heavy metal concentrations in vegetables (mg/kg, dry weight basis) ranged from 5.0 to 14.3 for Cu, 34.7 to 170 for Zn, 0.90 to 2.23 for Pb, and 0.45 to 4.1 for Cd around Dabaoshan mine in Guangdong, South China.

Suxian County, located in Hunan Province, South China, has a long history of mining activities dating back to the Jiaqing period of the Qing Dynasty. Zeng, *et al.* [10] reported that soil and waters were severely polluted by heavy metals near the Pb/Zn mine of Suxian County. Liao, *et al.* [11] concluded that the mean concentration of As in the soils from agricultural lands in Suxian County was 379.9 mg/kg. Similarly, Zhai, *et al.* [12] concluded that the mean concentration of Cd in these soils was 3.90 mg/kg. These values are far higher than China’s maximum permissible levels (MPLs) for soil [13]. The normal range for As in the soils of various countries was found to be 0.1 to 40 mg/kg (mean of 6 mg/kg) [14]. Desesso, *et al.* [15] indicated that As was likely to pose a risk to pregnant women and their offspring when soil As concentration was more than 100 mg/kg. The guideline for maximum recommended Cd intake set by WHO [16] and the United States Environmental Protection Agency (US EPA) [17] is 1 μg/(kg body weight·d). People are exposed to Pb, As, Cu and Cd mainly through food and drinking water (WHO) [17]. However, the health risks for a population exposed to heavy metals have rarely been evaluated in these regions; those evaluations actually conducted were limited to certain single-exposure routes such as vegetable consumption or inhalation [11,18]. Several studies have confirmed that there is a relationship between mortality and living near mining, smelting and processing areas [19,20,21,22]. Integrated health risk assessments for heavy metals through various exposure pathways are of significance for inhabitants living in mining areas.

The objectives of this paper are: (1) to quantify the concentrations of As, Pd, Cu and Cd in soil, rice, vegetable and dust; (2) to investigate the degree of pollution and the daily intake amount of toxic elements through soil, food and dust; (3) to present an integrated health risk assessment method for heavy metals through various exposure pathways for inhabitants living in a mining area.

## 2. Samples and Methods

### 2.1. Study Area

**Figure 1:**
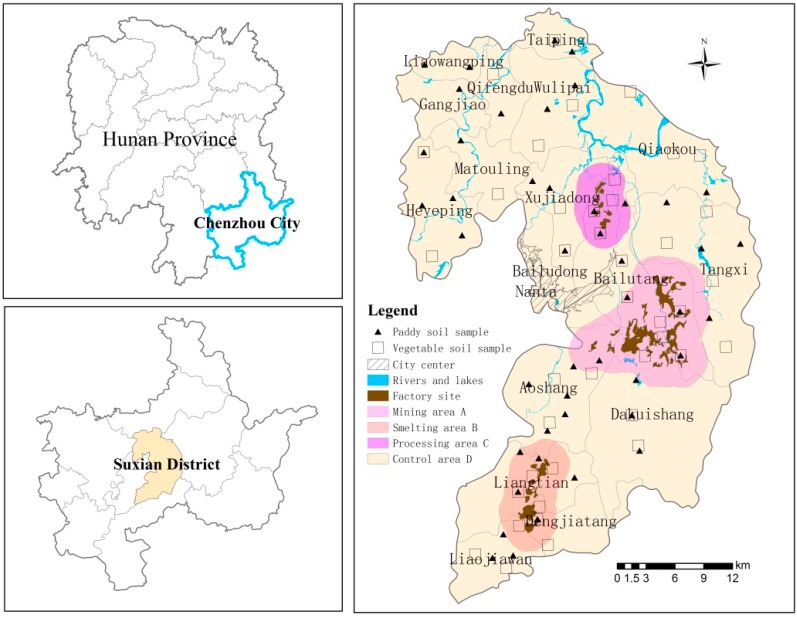
Illustration of sampling sites in Suxian County in Hunan Province, South China.

The study area, Suxian County (112°53′55″-113°16′20″E, 25°30′21″-26°03′29″N), is located in Chenzhou city, Hunan Province, South China (Figure 1). It lies in a typical subtropical humid monsoon climate zone with an annual average temperature of 18.2 °C and annual rainfall of 1487.2 mm (1981–2013). Rice is the main crop and a staple food, and pak choi, *Brassica campestris*, string bean, asparagus lettuce and capsicum serve as the main types of vegetables in the diets of the local residents. Suxian County has a 1000-year history of metal-mining activities and is characterized as a hilly and upland landscape. The investigated area comprises three functional areas as shown in the sampling map (Figure 1). Most of the largest mines in Suxian County, such as the Shizhuyuan, Dongbo and Manaoshan mines, are located around a cluster of mining sites (Mining Area A); the Dengjiatang smelter is located around the ore smelting site (Smelting Area B); and the Bailutang metals processing unit is located around a machining site (Processing Area C). The Shizhuyuan mine where Pb, Zn, W, Bi, Sn and Mo ores are mined and smelted is known as one of the largest metal mines in the world. 

### 2.2. Sampling

In our study, all soils sampled were from agricultural lands, including paddy and vegetable soils. Sample coordinates were obtained via the global positioning system. The samples were analyzed for heavy metal content. Six crop species were selected for this study: cereal (rice) and the vegetables, pak choi, *B. campestris*, string bean, asparagus lettuce and capsicum, which represent the major crop species growing in this area during the sampling season that included June and August of 2013. At each sampling site, samples were collected from the field by means of a random sampling method. Edible parts of crops and their rooted soil samples (at 0–15 cm in depth) were collected. Eighty-seven soil samples and two hundred and forty-five plant samples were collected in the districts. All samples were sealed in polyethylene bags. The spatial distribution of the sampling sites is shown in Figure 1.

### 2.3. Sample Preparation and Analysis

The soil samples were air-dried and ground to pass through a 100-micron mesh screen. The plant samples were washed with tap water to remove adhering soil, rinsed with de-ionized water, dried at 60 °C for 48 h in an oven, and then ground into fine powder. The soils were digested using HNO_3_-H_2_O_2_ [23]. The plants were digested using HNO_3_-HClO_4_ [24]. Samples were analyzed using inductively coupled plasma mass spectrometry (ICP-MS) and atomic absorption spectrophotometry (AAS). Standard reference materials for soils (GBW-07401) and plants (GBW-07602), obtained from the China National Center for Standard Reference Materials, were digested along with the samples and used for the Quality Assurance/Quality Control program. The precision and bias of the chemical analysis were less than 10%. All analyses were performed with the SPSS statistical package (v18.0) (SPSS Inc., Chicago, IL, USA).

### 2.4. Integrated Risk Assessment

#### 2.4.1. Daily Intake Estimate of Metals through Food

During the exposure assessment stage, an average daily intake dose (ADD, μg/(kg·d)) is used to quantify the oral exposure dosage for deleterious substances [25]. The daily intake amount of metals depends on both the heavy metal concentration and the amount of the respective food consumed. The ADD through consumption of food and soil can be calculated using the following formula:

(1) ADD = C × IR × ED × EF/(BM × AT)

The variables C, IR, ED, BM, EF and AT represent heavy metals content (μg/g), ingestion rate, exposure duration, reference body mass, exposure frequency and average time, respectively; the values of these parameters are listed in Table 1.

The average quantity of crops consumed by a person in China (adult/child) was chosen as 370/210 g/d of rice and 350/220 g/d of vegetables [26,27,28]. Furthermore, the average quantity of soil consumed by a person (adult/child) was chosen as 200/100 mg/d [26,29].

**Table 1:** Parameters used in risk assessment.

Ingestion rate (IR)	Value		Parameter	Value	
	Adult	Children		Adult	Children
**Soil**	150 mg	200 mg	Reference body mass (BM)	60 kg	25 kg
**Rice**	370 g	210 g	Exposure duration (ED)	365 d ^a^	365 d ^a^
**Vegetable**	350 g	220 g	Exposure frequency (EF)	74 a ^b^	74 a ^b^
**-**	**-**	**-**	Average time (AT)	27010 d ^c^	27010 d ^c^

Notes: ^a^, d is the abbreviation for day; ^b^, a is the abbreviation for age; ^c^, 27010 d = 365 d × 74 a.

#### 2.4.2. Human Health Risk Assessment

The hazard quotient (HQ) or hazard index (HI), a ratio of estimated exposure dose (ADD) and reference dose (RfD), characterizes the health risk of non-carcinogenic adverse effects due to exposure to toxicants [7,16]:

(2) HQ = ADD/RfD

(3) HI = ∑HQ

where RfD represents a toxicity index of a daily exposure to the population in comparison to a safe level of exposure orally over a lifetime. Thus, an index value < 1 is assumed to be safe over a lifetime. The health risks for local residents resulting from the intake of metals by consumption of crops and soil were assessed based on the index.

The maps of heavy metal spatial distribution in crop and soil were generated using ArcGIS v10.3 (Esri Corporation, USA). The data were analyzed using SPSS. The level of significance was set at *p* < 0.05. 

Table 2 shows the oral RfD for heavy metals in food [16,30,31].

**Table 2:** The oral reference dose (RfD) for heavy metals in food (mg/(kg·d)).

	As	Pb	Cu	Cd
**RfD**	3.00 × 10^−3^	3.50 × 10^−3^	4.00 × 10^−2^	1.00 × 10^−3^

**Table 3:** Heavy metal concentrations in soil samples. (mg/kg d.m.)

	Min	Max	Mean	Std
**As**	8.47	341.33	64.51	48.35
**Pb**	19.91	837.52	179.63	111.01
**Cu**	8.41	148.73	46.62	26.34
**Cd**	0.35	6.47	2.94	1.17

## 3. Results and Discussion

### 3.1. Concentrations of Heavy Metals in Soils

Heavy metal concentrations in the agricultural soils investigated in this study are shown in Table 3. A wide range of heavy metals concentrations (As: 8.47–341.33 mg/kg, Pb: 19.91–837.52 mg/kg, Cu: 8.41–148.73 mg/kg, Cd: 0.35–6.47 mg/kg) was found in the soil samples collected from various cultivated lands in Suxian County (Table 3). Compared with heavy metal concentrations in control soils (with mean values for As, Pb, Cu and Cd of 53.77 mg/kg, 64.34 mg/kg, 36.57 mg/kg, and 2.41 mg/kg, respectively), all heavy metals concentrations in the three functional areas were greatly elevated by the anthropogenic mining industry activities (Table 4). The highest concentrations of soil heavy metals were found at Mining Area A, where the mean concentrations of As, Pb, Cu and Cd were 244.25 mg/kg, 540.07 mg/kg, 111.03 mg/kg, and 5.72 mg/kg, respectively. The mean concentrations of heavy metals in functional areas B and C were also higher than those from control area D, by approximately 1.5–5 times.

The MPLs (maximum permissible levels) of soil heavy metals are generally used to assess the pollution level of As (30 mg/kg), Pb (300 mg/kg), Cu (100 mg/kg) and Cd (0.3 mg/kg) in agricultural soils. In all soil samples, As and Cd concentrations exceeding their MPLs were found in a significant proportion of the samples: approximately 32.5% and 41.2% of the soil samples, respectively, were found to contain As and Cd concentrations exceeding their Chinese MPLs [32]. The concentrations of the four heavy metals exceeded their MPLs according to Chinese standards for soil samples in all three functional areas. In contrast, within the scope of the control area, the vast majority of the sampled concentrations for the four heavy metals were lower than their respective Chinese MPLs standards.

**Table 4:** Mean concentrations of heavy metals in soils from different functional areas (mg/kg d.m.).

	As	Pb	Cu	Cd
**Mining Area A**	244.25	540.07	111.03	5.72
**Smelting Area B**	153.92	97.92	49.95	3.28
**Processing Area C**	170.84	315.29	93.99	5.06
**Control Area D**	53.77	64.34	36.57	2.41

**Figure 2:**
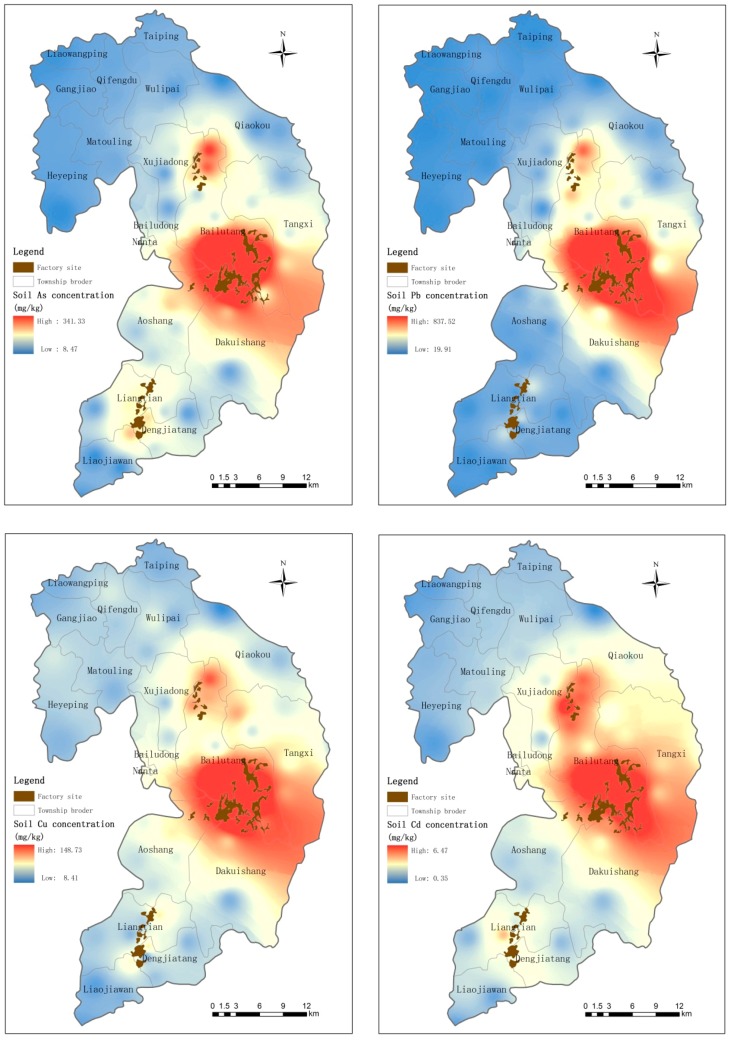
Geographical distribution of As, Pb, Cu and Cd levels in soils from the investigated area.

Samples of paddy and vegetable soils in different functional areas to one another were compared using the paired samples *T*-test and showed significant differences (*p* < 0.05) in soil As, Pb, Cu and Cd concentrations between the two land use types. On the basis of Geographic Information System (GIS) mapping of the spatial distributions of heavy metals in agricultural soils (Figure 1), three hotspot areas with high heavy metal concentrations were identified around the mine sites (Mining Area A), the metal processing sites (Processing Area C), and the smelting area (Smelting Area B) (Figure 2). Although the concentration of heavy metals in the vicinity of Area B is not as high as those around Areas A and C, compared to the control area (Area D), the concentration of heavy metals in this area is still in a higher range (Figure 2). Concentrations of heavy metals were found to decline with increasing distance from the mining area: the greatest soil heavy metal concentrations were found closest to Mining Area A (Figure 2). At the three functional areas, the mean concentrations of heavy metals decrease in the order: Area A > Area C > Area B.

Heavy metal concentrations in most samples collected from Area A, which has been associated with activities at the Shizhuyuan, Dongbo and Manaoshan mines at relatively larger scales and for longer periods, were higher than those from other the other mine industrial areas. At functional areas B and C, the regional ore smelters and metal processing plants are concentrated, and the plants are relatively small in size. However, due to the long-term mining and metallurgical activities at all three functional areas, significant difference were observed between the soil heavy metal concentrations at these areas *vs.* the control area, D.

The average heavy metal concentrations in soil samples collected throughout the Suxian area are relatively high, especially those in the vicinity areas of areas A, B and C; the concentrations of As and Cd in these areas are particularly high, followed by Pb and Cu. The maximum heavy metal concentrations of As and Cd in the soil are 10 times more than the stage **II** standard required by China Environmental Quality for Soils [32]. This illustrates that the soil in the range of the Suxian functional areas are contaminated mostly by As and Cd. Though the contamination mostly reflects the effect of the mining industry, it may also be related in part to the acidic soil (pH < 6.5) in the study area. At the same time, the concentration of Pb in the soil exceeds the 300 mg/kg standard required by China Environmental Quality for Soils [32]. The excess may also be related to the use of fertilizers and pesticides in the area.

### 3.2. Concentrations of Heavy Metals in Crops

The heavy metal concentrations in the measured crops are listed in Table 5 and Table 6.

**Table 5:** Heavy metal concentrations in crop samples (mg/kg d.m.).

	Rice					Asparagus lettuce					*B. campestris*			
	As	Pb	Cu	Cd		As	Pb	Cu	Cd		As	Pb	Cu	Cd
**Min**	0.02	0.66	0.09	ND		0.16	2.41	4.03	0.96		2.18	4.79	9.39	0.55
**Max**	1.48	5.78	6.75	1.39		10.7	33.5	21.6	6.91		18.9	41.8	50.5	7.38
**Mean**	0.39	2.01	2.37	0.23		3.42	10.6	9.23	2.23		5.87	13.4	20.2	2.39
**Std**	0.31	0.62	2.34	0.47		2.16	1.92	0.82	0.12		1.09	1.41	1.21	0.31
	Capsicum					String bean					Pak choi			
**Min**	0.06	1.39	3.52	0.28		0.14	1.55	2.43	0.18		1.03	3.11	9.17	1.82
**Max**	1.93	17.4	22.6	3.43		1.72	18.6	15.8	2.06		31.6	95.1	71.1	8.23
**Mean**	0.53	4.78	8.36	1.21		0.82	5.09	6.35	0.76		9.15	36.3	23.1	3.93
**Std**	0.42	2.19	4.24	0.31		0.22	1.13	1.86	0.34		4.53	15.45	11.45	1.41

**Table 6:** Mean heavy metal concentrations in crops from different functional areas (mg/kg d.m.).

	As			Pb			Cu			Cd	
	Rice	Vegetables		Rice	Vegetables		Rice	Vegetables		Rice	Vegetables
**Mining Area A**	1.07	9.13		4.27	30.84		1.12	27.73		4.32	3.89
**Smelting Area B**	0.40	3.27		1.99	12.96		0.40	16.62		1.91	2.45
**Processing Area C**	0.76	8.35		3.63	27.87		0.77	21.12		3.63	3.27
**Control Area D**	0.22	3.06		1.74	10.34		0.22	14.41		1.04	1.89

**Figure 3:**
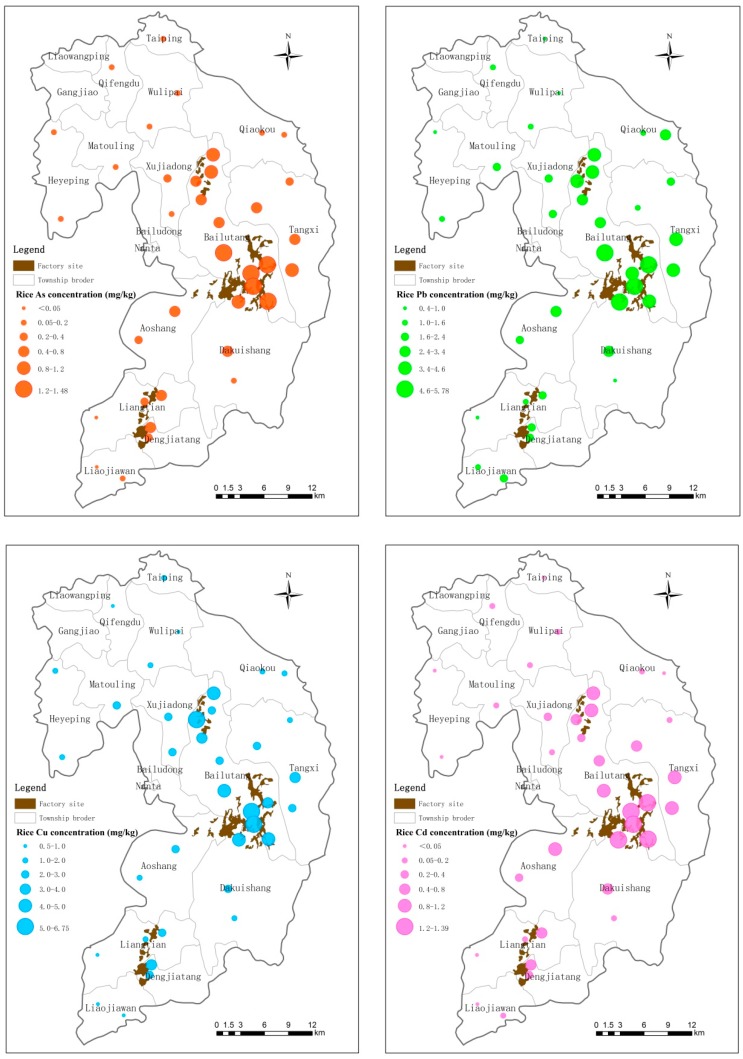
Concentrations of As, Pb, Cu and Cd in rice sampled from the investigated area.

Rice samples collected from the study area exhibited the following average heavy metal concentrations: As—0.39 mg/kg; Pb—2.01 mg/kg; Cu—2.37 mg/kg; and Cd—0.23 mg/kg. The highest concentrations of heavy metals were found in the vicinity of Mining Area A, where the mean concentrations of As, Pb, Cu and Cd are 1.07 mg/kg, 4.27 mg/kg, 1.12 mg/kg, and 4.32 mg/kg, respectively. The concentrations of heavy metals in brown rice samples collected at different functional areas were significantly different. Mining Area A and Processing Area C exhibited heavy metal concentrations that were significantly higher than those in Control Area D; the concentrations of heavy metals at Smelting Area B were slightly higher than those in the control area (Table 6 and Figure 3).

Obvious differences in heavy metal concentrations in the edible parts were found among the five vegetables tested. The mean As, Pb, Cu and Cd concentrations in the edible parts of asparagus lettuce, *B. campestris* and pak choi were higher than those of the other fruity vegetables. Heavy metal concentrations in the edible parts of all investigated vegetables followed the trend: pak choi > *B. campestris* > asparagus lettuce > capsicum > string bean. 

In the leafy vegetable samples, the highest heavy metal concentrations of As, Pb and Cd detected are higher than the MPLs allowed by the Sanitation Criterion for Food, China [33]. The As, Pb and Cd concentrations were 4.3, 3.6 and 4.3 times higher, respectively, than their permissible values, whereas the Cu concentration detected was slightly lower than its permissible value. As for fruity vegetables, the highest heavy metal concentrations detected for Pb and Cd are also higher (by 9.1- and 6.8-fold, respectively) than the MPLs allowed by the Sanitation Criterion for Food, China [33]. Similar to the spatial distribution of heavy metals in the soil, the plant samples that exceeded the permissible limits are located mostly near the three functional areas, particularly the town of Bailutang, which is near Mining Area A. The spatial distribution of heavy metals in vegetable samples indicates that the closer a sample is to a functional area, the higher the concentration of heavy metals can be found in it. Because China is faced with the scenario of a large population sharing limited land, it is a common phenomenon for residents living near mining functional areas to grow rice and vegetables in areas directly surrounding mines, resulting in heavy metal concentrations in food and vegetables higher than those in crops grown in unpolluted land.

Similar findings have also been reported in other parts of China. For example, Wang, *et al.* [34] analyzed the soil and plants around abandoned lead and zinc mines and showed that in radish edible parts, As and Pb, exceeded permissible levels by 5- to 220-fold, with As, Zn, Pb and Cu concentrations averaging 3.69 mg/kg, 73.23 mg/kg, 16.32 mg/kg and 62.20 mg/kg, respectively, in Shangyu city of Zhejiang province. Zhuang, *et al.* [35] reported that the concentrations of Cd and Pb in some food crop (rice grain, vegetable and soybean) samples were significantly higher than their MPLs in foods [33] in Chenzhou City in Southern China. Li, *et al.* [36] reported that rice and root vegetables were polluted severely and that the percentages of rice samples that exceeded heavy metal MPLs were 94.3%, 91.4%, 88.6%, and 17.1% for Pb, Cr, Cd, and Cu, respectively, in the Pearl River Estuary of China. Thus, crops grown near mining areas tend to contain excessive levels of heavy metals. Long-term consumption of such crops may bring great risks to human health. 

### 3.3. Daily Intakes via Various Exposure Pathways

Table 7 shows the mean daily estimated intake amounts of observed heavy metals in cereal (rice) and vegetables (pak choi, *B. campestris*, string bean, asparagus lettuce and capsicum) at each area. The daily dietary intake of As, Pb, Cu and Cd from food (rice and vegetables) varied from 7.75 × 10^−4^ to 3.23 × 10^−3^ mg/(kg·d), 1.33 × 10^−3^ to 3.58 × 10^−3^ mg/(kg·d), 1.65 × 10^−3^ to 3.46 × 10^−3^ mg/(kg·d) and 4.86 × 10^−4^ to 2.35 × 10^−3^ mg/(kg·d), respectively, for an adult in the investigated area. The mean concentrations for the daily dietary intake of As, Pb, Cu and Cd from food in the intensive mining area (A) were 4.17, 2.69, 2.09 and 4.83 times higher, respectively, than the mean concentrations of these heavy metals in food from control areas. The daily heavy metal intake from food varied greatly among different functional areas; residents from Mining Area A had the highest intake of heavy metals from both rice and vegetables.

**Table 7:** Daily dietary intake of heavy metals; exposure per day/(mg/(kg·d)).

	As		Pb		Cu		Cd	
	Adults	Children	Adults	Children	Adults	Children	Adults	Children
**Mining Area A**	3.23 × 10^−3^	4.91 × 10^−3^	3.58 × 10^−3^	5.66 × 10^−3^	3.46 × 10^−3^	5.38 × 10^−3^	2.35 × 10^−3^	3.61 × 10^−3^
**Smelting Area B**	1.13 × 10^−3^	1.73 × 10^−3^	1.44 × 10^−3^	2.27 × 10^−3^	1.48 × 10^−3^	2.33 × 10^−3^	1.11 × 10^−3^	1.69 × 10^−3^
**Processing Area C**	2.69 × 10^−3^	4.11 × 10^−3^	3.28 × 10^−3^	5.21 × 10^−3^	2.76 × 10^−3^	4.18 × 10^−3^	1.80 × 10^−3^	2.78 × 10^−3^
**Control Area D**	7.75 × 10^−4^	1.20 × 10^−3^	1.33 × 10^−3^	2.11 × 10^−3^	1.65 × 10^−3^	2.59 × 10^−3^	4.86 × 10^−4^	7.70 × 10^−4^

**Table 8:** Daily soil intake of heavy metals; exposure per day/(mg/(kg·d)).

	As		Pb		Cu		Cd	
	Adults	Children	Adults	Children	Adults	Children	Adults	Children
**Mining Area A**	8.52 × 10^−4^	2.72 × 10^−3^	1.78 × 10^−3^	5.69 × 10^−3^	3.69 × 10^−4^	1.18 × 10^−3^	3.47 × 10^−5^	1.31 × 10^−4^
**Smelting Area B**	5.52 × 10^−4^	1.76 × 10^−3^	7.76 × 10^−4^	2.48 × 10^−3^	1.48 × 10^−4^	4.75 × 10^−4^	1.64 × 10^−5^	5.13 × 10^−5^
**Processing Area C**	7.83 × 10^−4^	2.52 × 10^−3^	1.03 × 10^−3^	3.29 × 10^−3^	3.85 × 10^−4^	1.23 × 10^−3^	3.07 × 10^−5^	1.05 × 10^−4^
**Control Area D**	2.31 × 10^−4^	7.39 × 10^−4^	2.46 × 10^−4^	7.88 × 10^−4^	1.33 × 10^−4^	4.25 × 10^−4^	1.31 × 10^−5^	4.17 × 10^−5^

Table 8 indicates the mean daily estimated intake amounts of observed heavy metals in soils. The daily dietary intake of As, Pb, Cu and Cd from soils varied from 2.31 × 10^−4^ to 8.52 × 10^−4^ mg/(kg·d), 2.46 × 10^−4^ to 1.78 × 10^−3^ mg/(kg·d), 1.33 × 10^−4^ to 3.69 × 10^−4^ mg/(kg·d) and 1.31 × 10^−5^ to 3.47 × 10^−5^ mg/(kg·d), respectively, for an adult in the investigated area. For the ingestion of soils by children, the daily dietary intake of As, Pb, Cu and Cd from soils are significantly higher than those of adults, by approximately 3 times. Because children the amount of soil ingestion more than adults, and their weight lighter than adults.

The risk indices for As, Pb and Cd from food for residents in the mining and processing areas are greater than or close to 1, whereas those in the control areas with less mining activities are less than 1 (Table 9). The risk indices for As and Pb from soil for residents in the mining, smelting and processing areas are close to 1, whereas the risk indices for soil Cu and Cd over the whole study area are far lower than 1 (Table 10). The estimated exposures and risk indices for heavy metals demonstrate that there is an extremely high risk for adverse health effects from the consumption of rice and vegetables grown in the soil in functional areas where there are dense mining activities. The residents in the functional areas of Suxian County are potentially at risk for health problems resulting from food and soil consumption.

**Table 9:** Hazard quotients and risks for each heavy metal in crops.

	As		Pb		Cu		Cd	
	Adults	Children	Adults	Children	Adults	Children	Adults	Children
**Mining Area A**	1.076	1.638	1.025	1.618	0.086	0.135	2.353	3.610
**Smelting Area B**	0.378	0.576	0.411	0.651	0.036	0.058	1.109	1.693
**Processing Area C**	0.897	1.371	0.936	1.490	0.069	0.105	1.797	2.778
**Control Area D**	0.258	0.399	0.381	0.603	0.041	0.065	0.486	0.770

**Table 10:** Hazard quotients and risks for each heavy metal in soils.

	As		Pb		Cu		Cd	
	Adult	Children	Adult	Children	Adult	Children	Adult	Children
**Mining Area A**	0.283	0.906	0.507	1.623	0.009	0.030	0.035	0.131
**Smelting Area B**	0.183	0.586	0.222	0.710	0.004	0.012	0.016	0.051
**Processing Area C**	0.262	0.839	0.294	0.940	0.009	0.031	0.031	0.105
**Control Area D**	0.076	0.247	0.070	0.225	0.003	0.011	0.013	0.042

**Table 11:** Risk indices for crops and soils from different functional areas.

	Elements	THI (soil)		THI (food)		THI (total)	
		Adult	Children	Adult	Children	Adult	Children
**Control Area**	As	0.076	0.247	0.258	0.399	0.334	0.646
	Pb	0.070	0.225	0.381	0.603	0.451	0.828
	Cu	0.003	0.011	0.041	0.065	0.044	0.076
	Cd	0.013	0.042	0.486	0.770	0.499	0.812
**Functional Areas**	As	0.243	0.777	0.784	1.195	1.026	1.972
	Pb	0.341	1.091	0.791	1.253	1.132	2.344
	Cu	0.007	0.024	0.064	0.099	0.071	0.123
	Cd	0.027	0.093	1.753	2.694	1.780	2.787

As shown in Table 11, within the range of the study areas, the ingestion of four heavy metals through two routes, food and soil, contributes to the total non-carcinogenic risk index. The difference between functional areas and the control area is obvious; in the control area, the total hazard index (THI) values for non-carcinogenic risks from heavy metals in food and soil are all less than the safety threshold 1, and their sum is less than 1. This finding indicates that the potential health risks for residents in the control area are lower than those for residents in the functional areas, based on their dietary intake of heavy metals through food and soil. For the total population in the functional areas, the health risk evaluation index for children’s intake of Pb from soil reaches 1.091, which is greater than the safety threshold value of 1. For the ingestion of food by children, the THI values from As, Pb, and Cd are all greater than 1, having values of 1.195, 1.253 and 2.69, respectively. The THI value for an adult’s intake of the heavy metal Cd reaches 1.753, whereas the THI values for As and Pb come close to but do not exceed the safe threshold value of 1.

The THI analysis in Table 11 shows that for each of the four types of heavy metals, the total non-carcinogenic THI values for the control area are below 1. However, these THI values, particularly for the heavy metals As, Pb and Cd, have been trending toward the safe threshold value of 1. This means that if the pollution continues to be severe, the health risks for residents even in the control area may become dangerous. In the functional areas, there are already health risks to residents from As, Pb, and Cd, three types of heavy metals for which the THI values exceed 1. Previous data shows that children’s risk indices are higher than those for adults, so children face higher health risks. The order of severity of the heavy metal total non-carcinogenic risk is Cd > Pb > As > Cu. 

In China, some prior studies have shown that residents eating various vegetables will potentially incur major risks to their health through the intake of Pb and Cd contained in the vegetables; the risk to the health of children is higher than that for adults, and the risk for residents of mining areas is much higher than that for residents of a control area [37,38]. Other studies have shown that for residents in Japan and Korea, exposure to Cd primarily from a diet that is heavy in rice accounts for 40% and 23% of their total intake of Cd, respectively [39,40]. This indicates that arable land near mining areas is easily affected by mining; the surrounding soil can be polluted by sewage irrigation and falling dust.

Data from the United States Integrated Risk Information Database (US IRID) and WHO show that Pb can damage the brain and nervous system, causing neurological disorders and high blood pressure, and can lead to a slowing of growth in children, hearing impairment, headaches, reduction in learning ability, and abnormal behaviors [41]. The intake of As can cause cancer in internal organs (such as liver, kidney, lung, bladder), and can increase the risk for skin cancer [42]. With regard to the eating habits of residents in the study area, rice is the main cereal crop in Suxian County, and residents treat it as their staple food. The vast majority of local residents grow their own crops as a source of food in the study area, greatly increasing their health risks. However, because heavy metals have the characteristic that they tend to accumulate and persistent in an environment, the risk of other heavy metal contamination still exists. Related departments should pay increased attention to the situation, and they should take appropriate measures to address the problem of soil contamination and industrial dust emissions in Suxian County to reduce the harmful effects of heavy metals on people in the area, especially children. 

**Figure 4:**
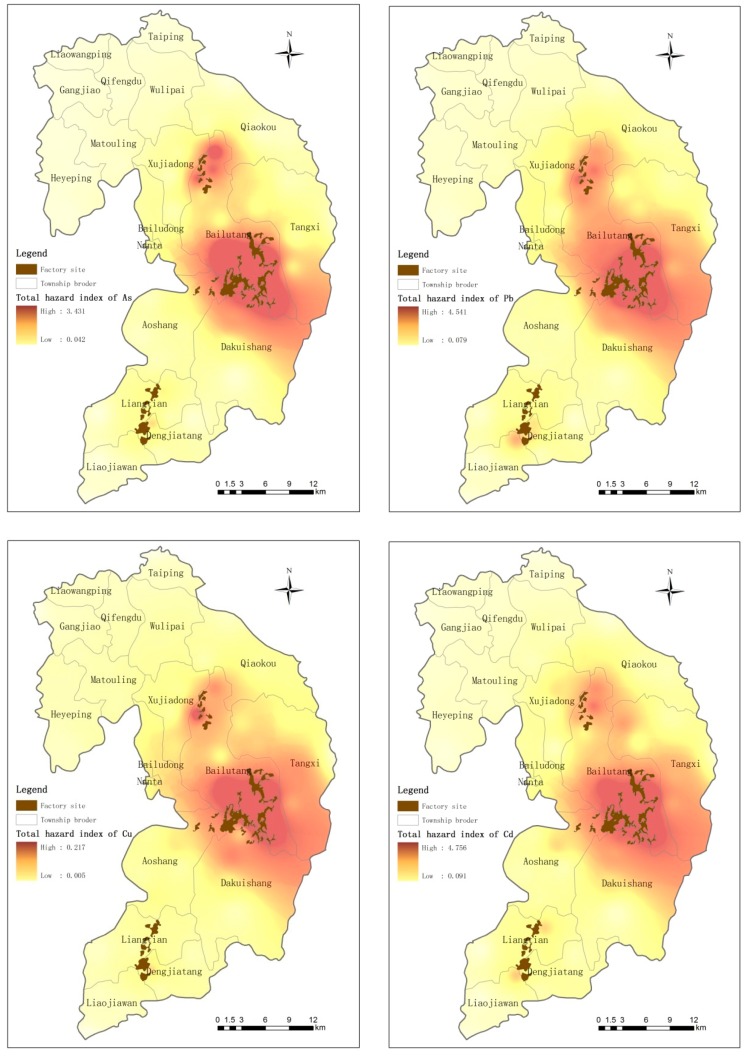
Geographical distribution of As, Pb, Cu and Cd non-carcinogenic risk indices in the study area.

Figure 4 shows the spatial distribution of the total non-carcinogenic risk of As, Pb, Cu and Cd. As shown in the figure, the non-carcinogenic risk index values for Suxian functional areas are obviously higher than those for the control area, and different functional areas have different health risk levels. In order of decreasing health risk, the areas are Area A > Area C > Area B. 

## 4. Conclusions

The results of this study indicate that the heavy metal concentrations in the soil and crops vary significantly in different functional regions of the study area. In particular, soil and crop samples in the three functional areas exhibit As, Pb and Cd concentrations in higher ranges relative to the control area. The mean concentrations of As, Cd and Pb in the soils still slightly exceed the corresponding MPLs for agricultural soils in China. Heavy metal contents also differ between different functional areas, with the highest concentrations in Mining Area A, followed by Processing Area C, then Smelting Area B. Soil heavy metal concentrations abruptly decrease with increasing distance from the pollutant source. Compared with their concentrations in the control area, As, Pb, Cu and Cd concentrations in rice and vegetables in the functional areas were found to be remarkably high. Heavy metal concentrations in the edible parts of leafy vegetables are substantially higher than those in non-leafy vegetables. Thus, non-leafy vegetables are recommended for cultivation in the contaminated soils. 

The estimated ADD of the considered toxic elements (As, Pb, Cu and Cd) for residents via oral ingestion includes food consumption and soil ingestion. Residents’ ADD of As, Pb and Cd have exceeded the recommended dietary allowance levels within the three functional areas of the study area. Long-term As, Pb and Cd exposure by regular consumption of the soil, rice and vegetables in the investigated area pose potential health problems to residents in the vicinity of the mining industry. Therefore, greater attention should be paid to the potential health risks posed by the consumption of local crops with high heavy metal concentrations and the ingestion/inhalation of contaminated soils around industrial districts.

## References

[1] Benin A., Sargent J. (1999). High concentrations of heavy metals in neighborhoods near ore smelters in northern Mexico. Environ. Health Perspect..

[2] Taylor H., Appleton J.D., Lister R., Smith B., Chitamweba D., Mkumbo O., Machiwa J.F., Tesha A.L., Beinhoff C. (2005). Environmental assessment of mercury contamination from the Rwamagasa artisanal gold mining centre, Geita District, Tanzania. Sci. Total Environ..

[3] Liu H.Y., Probst A., Liao B.H. (2005). Metal contamination of soils and crops affected by the Chenzhou lead/zinc mine spill (Hunan, China). Sci. Total Environ..

[4] Obiri S. (2007). Determination of heavy metals in water from boreholes in Dumasi in the Wassa westdistrict of western region of Republic of Ghana. Environ. Monit. Assess..

[5] Marsh G.M., Esmen N.A., Buchanich J.M., Youk A.O. (2009). Mortality patterns among workers exposed to arsenic, cadmium, and other substances in a copper smelter. Am. J. Ind. Med..

[6] Sun H.F., Li Y.H., Ji Y.F., Yang L.S., Wang W.Y., Li H.R. (2010). Environmental contamination and health hazard of lead and cadmium around Chatian mercury mining deposit in western Hunan Province, China. Trans. Nonferrous Metal. Soc. China.

[7] World Health Organization (WHO) (1993). Evaluation of Certain Food Additives and Contaminants (41st Report of the Joint FAO/WHO Expert Committee on Food Additives).

[8] World Health Organization (WHO) (2001). Codex Maximum Level for Cadmium in Cereals. Pulses and Legumes.

[9] Zhuang P., Zou B., Li N.Y., Li Z.A. (2009). Heavy metal contamination in soils and food crops around Dabaoshan mine in Guangdong, China: implication for human health. Environ. Geochem. Health.

[10] Zeng Q.R., Yang R.B., Zhou X.H., Tie B.Q. (1995). Characteristics of their fractionation in the area polluted by the heavy metals of lead-zinc ore tailing particulates. Hunan Agr. Sci..

[11] Liao X.Y., Chen T.B., Xie H., Liu Y.R. (2005). Soil As contamination and its risk assessment in areas near the industrial districts of Chenzhou City, Southern China. Environ. Int..

[12] Zhai L.M., Liao X.Y., Chen T.B., Yan X., Xie H., Wu B., Wang L. (2008). Regional assessment of cadmium pollution in agricultural lands and the potential health risk related to intensive mining activities: A case study in Chenzhou City, China. J. Environ. Sci..

[13] National Environmental Protection Agency of China (NEPAC) (1995). Environmental Quality Standard for Soils.

[14] Chatterjee A., Das D., Chakraborti K. (1993). A study of groundwater contamination by arsenic in the residential area of Behala, Calcutta, due to industrial pollution. Environ. Pollut..

[15] Desesso J.M., Jacobson C.F., Scialli A.R., Farr C.H., Holson J.F. (1998). An assessment of the developmental toxicity of inorganic arsenic. Reprod. Toxicol..

[16] United States Environmental Protection Agency (US EPA) (2000). Risk-Based Concentration Table.

[17] World Health Organization (WHO) (2009). Towards an Assessment of the Socioeconomic Impact of Arsenic Poisoning in Bangladesh: Health Effects of Arsenic in Drinking Water.

[18] Zheng N., Wang Q., Zheng D. (2007). Health risk of Hg, Pb, Cd, Zn, and Cu to the inhabitants around Huludao Zinc plant in China via consumption of vegetables. Sci. Total Environ..

[19] Pan J., Plant J.A., Voulvoulis N., Oates C.J., Ihlenfeld C. (2010). Cadmium levels in Europe: Implications for human health. Environ. Geochem. Health.

[20] Wang M., Xu Y., Pan S., Zhang J., Zhong A., Song H., Ling W. (2011). Long-term heavy metal pollution and mortality in a Chinese population: An ecologic study. Biol. Trace Elem. Res..

[21] Hawkesworth S., Wagatsuma Y., Kippler M., Fulford A.J.C., Arifeen S.E., Persson L.A., Moore S.E., Vahter M. (2013). Early exposure to toxic metals has a limited effect on blood pressure or kidney function in later childhood, rural Bangladesh. Int. J. Epidemiol..

[22] Song D.P., Jiang D., Wang Y., Chen W., Huang Y.H., Zhuang D.F. (2013). Study on Association between Spatial Distribution of Metal Mines and Disease Mortality: A Case Study in Suxian District, South China. Int. J. Environ. Res. Public Health.

[23] Chen T.B., Fan Z.L., Lei M., Huang Z.C., Wei C.Y. (2004). Effect of phosphorus on arsenic uptake by As-hyperaccumulat or *Pterisvittata* L. and its implication. Chin. Sci. Bull..

[24] Liao X.Y., Chen T.B., Xie H., Xiao X.Y. (2004). Effect of application of P fertilizer on efficiency of As removal from As contaminated soil using phytoremediation: field study. Acta Sci. Circumstantiae.

[25] Tripathi R.M., Raghunath R., Krishnamoorthy T.M. (1997). Dietary intake of heavy metals in Bombay city, India. Sci. Total Environ..

[26] Stanek E.J., Calabrese E.J. (1995). Daily estimates of soil ingestion in children. Environ. Health Perspect..

[27] Wang X.L., Sato T., Xing B.S., Tao S. (2005). Health risks of heavy metals to the general public in Tianjin, China via consumption of vegetables and fish. Sci. Total Environ..

[28] Feng Z.M., Shi D.F. (2006). Chinese food consumption and nourishment in the latest 20 years. Resour. Sci..

[29] Edware J.C., Ramon B., Penelope P. (1997). Soil ingestion in adults results of a second pilot study. Ecotoxicol. Environ. Safety.

[30] United States Environmental Protection Agency (US EPA) (1989). Risk Assessment Guidance for Superfund, Vol. I: Human Health Evaluation Manual.

[31] United States Environmental Protection Agency (US EPA) (2002). Supplemental Guidance for Developing Soil Screening Levels for Superfund Sites.

[32] National Environmental Protection Agency of China (NEPAC) (1995). Environmental Quality of Standard for Soils.

[33] National Environmental Protection Agency of China (NEPAC) (2005). Maximum Levels of Contaminants in Foods.

[34] Wang Y., Zhao Q.L., Hu Y. (2011). Survey and contamination assessment of heavy metals in soil and plants around the Pb/Zn mine in Shangyu, Zhengjiang province. Environ. Chem..

[35] Zhuang P., Lu H.P., Li Z.A., Zou B., McBride M.B. (2014). Multiple exposure and effects assessment of heavy metals in the population near mining area in south China. PLoS ONE.

[36] Li Q., Chen Y., Fu H., Cui Z., Shi L., Wang L., Liu Z. (2012). Health risk of heavy metals in food crops grown on reclaimed tidal flat soil in the Pearl River Estuary, China. J. Hazard. Mater..

[37] Sun Q.B., Yin C.Q., Deng J.F., Zhang D.F. (2013). Characteristics of soil-vegetable pollution of heavy metals and health risk assessment in Daye mining area. Environ. Chem..

[38] Yang S.X., Yi L.B., Liu J., Wang H., Suo Y.Y. (2012). Heavy Metals Concentrations and Health Risk in Vegetables Grown on Mn and Pb/Zn Mineland in Huayuan County, West Hunan Province, China. J. Agro. Environ. Sci..

[39] Nakadaira H., Nishi S. (2003). Effects of low-dose cadmium exposure on biological examinations. Sci. Total Environ..

[40] Moon C.S., Zhang Z.W., Shimbo S., Watanabe T., Moon D.H., Lee C.U., Lee B.K., Ahn K.D., Lee S.H., Ikeda M. (1995). Dietary intake of cadmium and lead among the general population in Korea. Environ. Res..

[41] United States Environmental Protection Agency (US EPA) (2004). Lead and Compounds (Inorganic).

[42] United States Environmental Protection Agency (US EPA) (1993). Arsenic, Inorganic.

